# Endothelial progenitor cells and circulating microRNAs as possible biomarkers for coronary artery disease

**DOI:** 10.3389/fragi.2026.1850689

**Published:** 2026-07-15

**Authors:** Jakub Jozue Wojtacha, Jan Budzianowski, Magdalena Wojciech, Edyta Wawrzyniak-Gramacka, Barbara Morawin, Jarosław Hiczkiewicz, Agnieszka Zembron-Lacny

**Affiliations:** 1 Department of Applied and Clinical Physiology, Collegium Medicum, University of Zielona Gora, Zielona Góra, Poland; 2 Department of Interventional Cardiology and Cardiac Surgery, Collegium Medicum, University of Zielona Gora, Zielona Góra, Poland; 3 Department of Applied Mathematics, Institute of Mathematics, Faculty of Exact and Natural Sciences, University of Zielona Góra, Zielona Góra, Poland

**Keywords:** aging, coronary syndrome, endothelial progenitor cells, inflammation, nitric oxide, oxidized LDL

## Abstract

**Introduction:**

Endothelial cells and their precursors, i.e., endothelial progenitor cells (EPCs), along with other cell types such as smooth muscle cells and immune cells, release or sequester microRNAs (miRNAs) that actively participate in endothelial dysfunction, smooth muscle cell proliferation, vascular inflammation, and progression of atherosclerotic plaques, ultimately contributing to the transition from stable to acute coronary syndromes. The stability of circulating miRNAs has, for many years, attracted interest in their use as biomarkers in the diagnosis and monitoring of coronary artery disease (CAD).

**Methods:**

The study was designed to assess the association of total circulating miRNAs with endothelial dysfunction and EPC-related parameters. The study was carried out in seventy-two patients with CAD (73.2 ± 5.6 years). They were compared with eighty healthy controls (HC) (70.9 ± 5.4 years).

**Results:**

The lipid-lipoprotein profile including triglycerides, total cholesterol, low-density lipoprotein (LDL), high-density lipoprotein (HDL), non-HDL and oxidized LDL as well as endothelium-specific variables such as nitric oxide, 3-nitrotyrosine, early EPCs and total EPCs were reduced in CAD patients (p <0.001). The lower levels of nitric oxide in CAD patients (77.60 ± 49.58 μmol/L) compared to HC (290.64 ± 151.93 μmol/L) confirmed the impairment of endothelial secretory function. Total circulating miRNA levels were three-fold higher in CAD (9.36 ± 1.48 ng/L) than in HC (3.22 ± 0.48 ng/L). For miRNA, the area under the curve (AUC) was 1.0 (sensitivity 100%, specificity 100%) with a cut-off value of 5.37 ng/L, indicating a strong discriminatory potential between CAD patients and healthy controls. However, the diagnostic performance of total circulating miRNAs (AUC = 1.0) was observed in a relatively small, single-centre cohort and should be considered exploratory, requiring confirmation in larger, independent studies.

**Discussion:**

Our study provides further evidence of complex interactions between EPCs and total circulating microRNAs in coronary artery disease. The findings suggest that circulating microRNAs, particularly in combination with EPC-related parameters, may represent promising biomarkers for clinical differentiation and characterization of endothelial dysfunction in CAD patients.

## Introduction

Age plays a significant role in the deterioration of cardiovascular functionality, resulting in an increased risk of cardiovascular disease (CVD). CVD affects 70%–80% of individuals aged 60%–79% and 85% of those aged >80 years. Common conditions include hypertension, coronary artery disease, heart failure, and stroke which often coexist with other comorbidities such as diabetes, arthritis, and frailty, thereby significantly increasing the cardiovascular risk (Forouzandeh et al., 2024). Sex differences are also frequently observed in older adults, with regard to both the onset and prevalence of CVD (Rodgers et al., 2019). In the AHA 2025 Heart Disease and Stroke Statistical Update, the incidence of CVD was reported to be 77.8% in males and 76.3% in females, among individuals aged 60–79 years. Furthermore, the incidence of CVD was recorded at 85%–90% in patients aged >80 years with a slightly higher prevalence observed in females than in males (Moscucci et al., 2026). With respect to coronary artery disease (CAD), the strongest risk factors include increasing age and male gender (Nazli et al., 2024).

Endothelial cells, along with other cell types such as smooth muscle cells and immune cells, release or sequester microRNAs (miRNAs) that contribute to endothelium dysfunction in patients suffering from CAD (Bouley et al., 1975; Chamorro-Jorganes et al., 2013). Currently, two main hypotheses describe the role of endothelial progenitor cells (EPCs) in vascular repair. The first one suggests that EPCs can directly differentiate into endothelial cells and integrate into injured vessels, whereas the second proposes that EPCs affect the cells and blood vessels by releasing signaling molecules including miRNAs. Today, increasing evidence supports the latter mechanism (Yan et al., 2022; Doleschall et al., 2025). Therefore, the secretory properties of EPCs warrant further studies. EPCs are typically defined retrospectively by their ability to differentiate into endothelial cells, however, no universally accepted molecular definition exists. They are determined by cell surface markers, including CD34, CD133/CD34, CD133/vascular endothelial growth factor receptor-2 or CD133/CD34/vascular endothelial growth factor receptor-2. EPCs are currently classified in two main populations: early endothelial progenitor cells (eEPCs) and late outgrowth endothelial progenitor cells. The former participate in revascularization mainly through a paracrine mechanism by the secretion of several factors including vascular endothelial growth factor (VEGF), interleukin-2 (IL-2), interleukin-8 (IL-8), granulocyte colony-stimulating factor (G-CSF), granulocyte–macrophage colony-stimulating factor (GM-CSF), hepatocyte growth factor (HGF), and interleukin-10 (IL-10) which promote the activity of endothelial colony forming cells and other vascular cells (Doleschall et al., 2025). In contrast to eEPCs, EPCs are considered the “true” endothelial progenitor cells, due to their capacity for revascularization by direct incorporation in the newly formed vessels *in vivo* (Zhang et al., 2022). Both early and mature/true endothelial progenitor cells express endothelial nitric oxide synthase (eNOS) and are highly sensitive to misbalances between nitric oxide (NO) and superoxide (O_2_^−).^ Production (Fleissner and Thum, 2011). Multiple studies have shown that the cardiovascular risk factors accelerate the numerical exhaustion and senescence of EPCs, however, the molecular mechanisms governing these changes remain poorly understood EPCs depletion may be related to increased oxidative stress and systemic inflammation, both of which contribute to impaired vascular repair and the expansion of endothelial damage (Tomić et al., 2025).

Endothelial progenitors release miRNAs that contribute to multiple cytophysiological processes including the regulation of autophagy in EPCs, the modulation of EPCs differentiation and their involvement in thrombus resolution (Du et al., 2020). From a methodological perspective, circulating miRNAs can be reliably detected in serum and plasma with high sensitivity and specificity (Pozniak et al., 2022). Although free miRNAs are susceptible to degradation by RNases, a substantial proportion of circulating miRNAs is protected through binding to high-density lipoproteins or encapsulation within extracellular vesicles, including exosomes and apoptotic bodies, thereby conferring remarkable stability in the circulation (Komal et al., 2019). These properties make circulating miRNAs particularly attractive candidates for clinical biomarker development.

Recent comprehensive reviews have further reinforced the central role of miRNAs in CAD, demonstrating that dysregulated microRNA networks actively participate in endothelial dysfunction, smooth muscle cell proliferation, vascular inflammation, and progression of atherosclerotic plaques, ultimately contributing to the transition from stable CAD to acute coronary syndromes (Mansour et al., 2025). Collectively, these observations provide strong biological plausibility for the relevance of miRNAs in the pathophysiology of CAD and support their emerging diagnostic and therapeutic potential. Many miRNAs exhibit altered expression levels in CAD patients and can help distinguish CAD patients from healthy individuals (Melak and Baynes, 2019). In recent years, there has also been an increasing interest in circulating miRNAs as diagnostics markers across different phases of CAD (Pozniak et al., 2022; Melak and Baynes, 2019). Over 50 miRNAs have been identified as critical regulators of vascular homeostasis and CAD progression, playing significant roles in atherosclerotic plaque development, endothelial cell senescence, and impaired vascular repair. Furthermore, circulating miRNAs have emerged as promising low-cost and non-invasive biomarkers for early detection, risk assessment, and therapeutic targeting in CAD (Mansour et al., 2025). In this context, both EPCs and miRNAs are currently considered as potential biomarkers of vascular homeostasis and cardiovascular risk prognosis (Benítez-Camacho et al., 2023), although a deeper understanding of their implications in cardiovascular complications is still needed. Several studies have suggested that a reduced number of EPCs in peripheral blood is associated with an increased risk of CAD progression (Tomić et al., 2025). Therefore, a combined analysis of endothelial progenitor cells together with miRNAs can provide a more profound insight into the most relevant processes involved in CAD to improve prediction and prevention of vascular diseases development.

## Materials and methods

### Study population

The study included seventy-two patients aged 61–85 years (males *n* = 43, females *n* = 29) admitted to the Department of Cardiology in Multidisciplinary Hospital in Nowa Sol due to CAD (Table 1). The diagnoses of unstable angina (UA) and chronic coronary syndromes (CCS) were established according to the current European Society of Cardiology (ESC) guidelines, based on clinical presentation and results of appropriate diagnostic tests (ESC Scientific Document Group, 2023; Vrints et al., 2024). Among the study participants, 28 patients (*n* = 28) were diagnosed with UA, whereas the remaining 44 patients (*n* = 44) had CCS. All patients with UA underwent coronary angiography, and percutaneous coronary intervention (PCI) was performed in 12 of them. In the CCS group, 7 patients underwent coronary angiography alone, while 4 patients underwent PCI. The remaining patients with CCS were treated conservatively. Current smokers were excluded from the study. Data on pharmacological therapy in the study group were collected and are summarized in Table 2. The CAD group was compared to eighty healthy controls (HC) aged 70.9 ± 5.4 years (males *n* = 18, females *n* = 62). The current health status of the control group was assessed on the basis of medical records at a routine follow-up visit to a primary care physician. On the basis of the medical interview the following exclusion criteria were applied: acute infectious and autoimmunological diseases, uncontrolled hypertension and/or diabetes, oncologic diseases and neurodegenerative diseases. Individuals with a history of cardiovascular events were also excluded from the study. The medications taken by the healthy controls included antihypertensive (84%) and hypolipidemic (10%) drugs as well as anticoagulants including anti-platelet agents (15%). All individuals were informed of the aim of the study and signed a written consent to participate in the project. The study design received approval from the Bioethics Commission at University Zielona Gora (No. UZ9/2023). The study adhered to the principles and standards outlined in the Declaration of Helsinki and the Guidelines for Good Clinical Practice.

**TABLE 1:** Basic characteristics of study sample.

Variables	CAD *n* = 72 Mean ± SD (Me)		HC *n* = 80 Mean ± SD (Me)		CAD vs. HC *p* level
	Male *n* = 43	Female *n* = 29	Male *n* = 18	Female *n* = 62	
Age (yr)	72.7 ± 5.8 (73.0)	74.0 ± 5.3 (73.0)	73.9 ± 5.5 (72.0)	70.0 ± 4.9 (70.5)	0.011
Body mass (kg)	92.4 ± 17.3 (90.0)	75.7 ± 14.7 (74.0)	80.0 ± 8.4 (75.6)	65.7 ± 6.7 (65.3)	<0.001
Height (cm)	173.2 ± 6.2 (174.0)	161.9 ± 6.2 (162.0)	168.5 ± 4.7 (168.6)	159.7 ± 5.1 (159.4)	<0.001
BMI (kg/m^2^)	30.7 ± 4.8 (29.6)	29.0 ± 5.7 (28.5)	27.9 ± 2.6 (27.9)	26.1 ± 3.0 (25.9)	<0.001

Abbreviations: CAD, coronary artery disease; HC, healthy controls; BMI, body mass index; SD, standard deviation; Me, median.

**TABLE 2:** Treatment of coronary artery disease patients.

Therapy	CAD *n* = 72
Antiplatelets agents	50.7%
Anticoagulants	42.5%
Beta blockers	78.1%
Angiotensin-converting enzyme inhibitors	53.4%
Angiotensin receptor blocker	21.9%
Lipid-lowering agents	100%
Additional treatment options	100%

Abbreviations: CAD, coronary artery disease.

### Blood samples collection

Fasting blood samples were collected in patients with CAD on admission for a routine follow-up visit using S-Monovette tubes (Sarstedt AG & Co. KG, Nümbrecht, Germany). The whole blood samples were placed into specimen tubes containing EDTA and were immediately analyzed. For the other biochemical analyses blood samples were centrifuged at 3,000 rpm for 10 min, and aliquots of serum were stored at −80 °C.

### White blood cell count-derived inflammation indices

The total white blood cell (WBC) count, platelet count and differential white blood cell counts were determined by Sysmex XN-1000 (Sysmex Europe Gmbh, Norderstedt, Germany). The pan-immune-inflammation value (PIV) was calculated according to the following formula: PIV = [neutrophil count (10^3^/µL) × platelet count (10^3^/µL) × monocyte count (10^3^/µL)]/lymphocyte count (10^3^/µL) proposed by Fucà et al. (2020). The neutrophils to high density lipoproteins ratio (NHR), as an integrated marker of inflammation and dyslipidemia, was calculated as neutrophils count (10^3^/µL) divided by high-density lipoproteins (HDL) level (mg/dL) (Ren et al., 2023).

### Lipoprotein-lipid profile and C-reactive protein

Serum triglycerides (TG), total cholesterol (TC), HDL and low-density lipoproteins (LDL) were determined using BM200 Biomaxima (Poland). The non-HDL cholesterol was calculated by subtracting HDL from the total cholesterol concentration. Oxidized low-density lipoprotein (oxLDL) was determined using ELISA kits from SunRed Biotechnology Company (Shanghai, China) with detection limit at 30.3 ng/mL. Serum C-reactive protein (CRP) was measured using a high sensitivity commercial ELISA kit from DRG International (Springfield Township, Cincinnati, OH, United States) with the detection limit of 0.001 mg/L.

### Endothelium-specific variables

Nitric oxide (NO), 3-nitrotyrosine (3NT), peripheral blood mononuclear cells with surface marker CD34 (early endothelial progenitor cells, eEPCs) and with surface marker endothelin-1 (total endothelial progenitor cells, EPCs) were determined by using ELISA kits from SunRed Biotechnology Company (Shanghai, China) with detection limits 2.052 mol/L, 0.007 nmol/mL, 0.167 ng/mL and 0.125 ng/mL, respectively. The average intra-assay coefficients of variation (intra-assay CV) for the used enzyme immunoassay tests (ELISA) were <8%. The total circulating miRNA levels were measured directly in serum using a Quant-iTTM RNA high-sensitivity assay kit and a Qubit fluorometer (Invitrogen, Carlsbad, CA, United States) in accordance with the manufacturer’s instructions. The samples were analyzed in duplicate, and the mean of the two measurements was used as the final value. The intra-assay CV for the Quant-iTTM RNA high-sensitivity assay was <2%.

### Statistical analysis

Statistical analyses were performed using R 4.2.1 software [R Core Team. R: A language and environment for statistical computing. R Foundation for Statistical Computing, Vienna, Austria. URL (2022); https://www.R-project.org/]. The variables were reported as mean values ±standard deviation (SD) and median (Me). The assumptions for the use of parametric or nonparametric tests were checked using the Shapiro-Wilk and Levene’s tests to assess the normality of the distributions and the homogeneity of variances, respectively. The significant differences in mean values between the groups were evaluated by the Student’s t-test. If the normality and homogeneity assumptions were violated, the Mann-Whitney nonparametric test was used. Spearman’s rank correlation (r_s_ Spearman’s rank correlation coefficient) was used to investigate the relationships between inflammatory and endothelial variables. The predictive value of inflammatory and endothelial variables was evaluated using the receiver operating characteristic curve (ROC). Area under the ROC Curve (AUC) was used to provide an aggregate measure of performance across all possible classification thresholds. Both univariate and multivariate logistic regression models were used. The optimal threshold value for clinical stratification (cut-off value) was obtained by calculating the Youden index. Statistical significance was set at *p* < 0.05.

## Results

### Study population

The BMI values were significantly higher in CAD patients than in healthy elderly. The study patients demonstrated body mass index (BMI) ranging from 17 to 45.3 kg/m^2^, whereas in HC the values ranged from 20.7 to 32.5 kg/m^2^. Only 16% of the study patients were normal weight (18.5–24.9 kg/m^2^) whereas 44% were classified as overweight (25.0–29.9 kg/m^2^) and 40% as obese (≥30 kg/m^2^) (Table 1). Despite the absence of differences in lipoprotein-lipid profile between normal-weight and overweight patients, pan-immune-inflammation value was significantly higher in patients with BMI >30 kg/m^2^ (PIV 558 ± 563) compared with those with BMI <25 kg/m^2^ (PIV 338 ± 142). Obese patients demonstrated a marked endothelial dysfunction which was manifested by a low NO level of 64 ± 41 μmol/L, whereas patients with normal weight had NO of 97 ± 67 μmol/L. A similar trend was observed for endothelial progenitor cells, i.e., obese patients had EPCs of 7.40 ± 8.71 ng/mL, compared with EPCs of 15.69 ± 19.63 ng/mL in normal-weight patients. However, no corresponding relationship was observed for total circulating miRNA.

### White blood cell count-derived inflammation indices

The total white blood cells, platelets and differential white blood cell counts fell within the referential range in all our study patients (Table 3). Neutrophils and monocytes tended toward high values in coronary syndrome whereas platelet and lymphocyte counts did not differ between groups, which resulted in an increase (*p* < 0.001) in PIV (477 ± 447) in coronary syndrome compared to healthy individuals (222 ± 166). The difference was particularly pronounced in males. The PIV in men with CAD was 568 ± 506, while in healthy men it was 209 ± 121. NHR, as an integrated marker of inflammation and dyslipidemia, was two-fold higher in CAD patients (0.095 ± 0.037) than HC group (0.040 ± 0.019). There were no differences in NHR between females and males in either the CAD or HC groups.

**TABLE 3:** White blood cell count-derived inflammation indices.

Variables	Reference values	CAD *n* = 72		HC *n* = 80		CAD vs. HC *p* level
		Mean ± SD	Me (IQR 25%–75%)	Mean ± SD	Me (IQR 25%–75%)	
WBC (10^3^/µL)	4.0–10.2	7.63 ± 2.14	7.16 (6.29–9.02)	5.97 ± 1.63	5.68 (4.94–6.78)	<0.001
Neutrophils (10^3^/µL)	2.0–6.9	4.97 ± 1.90	4.65 (3.88–6.12)	3.27 ± 1.19	3.03 (2.50–3.84)	<0.001
Lymphocytes (10^3^/µL)	0.6–3.4	1.93 ± 1.13	1.78 (1.31–2.23)	1.92 ± 0.66	1.83 (1.56–2.18)	0.409
Monocytes (10^3^/µL)	0.00–0.90	0.66 ± 0.25	0.61 (0.49–0.78)	0.52 ± 0.16	0.50 (0.42–0.59)	<0.001
Platelets (10^3^/µL)	140–420	223 ± 70	211 (174–277)	222 ± 50	224 (195–254)	0.995

Abbreviations: CAD, coronary artery disease; HC, healthy controls; WBC, white blood cells; WBC, white blood cells; SD, standard deviation; Me, median; IQR, interquartile.

### Lipoprotein-lipid profile and C-reactive protein

The total cholesterol and lipoproteins have been proven to be the strongest biomarkers of vascular diseases. The significantly lower levels of TG, TC, LDL, HDL and non-HDL were found in patients with CAD likely reflecting the effects of lipid-lowering therapy and standard cardiovascular treatment. Regardless of the therapy used, CRP levels were approximately two-fold higher in CAD patients (6.66 ± 3.72 mg/L) compared to HC (3.16 ± 3.12 mg/L). OxLDL did not differ between groups but it highly correlated with endothelium-specific variables, including NO (r_s_ = 0.878, *p* < 0.001) and 3NT (r_s_ = 0.773, *p* < 0.001) in patients with CAD. There were no differences in lipoprotein-lipid profile (Table 4) and CRP levels between females and males in either the CAD or HC groups.

**TABLE 4:** Lipoprotein-lipid profile.

Variables	Reference values*	CAD *n* = 72		HC *n* = 80		CAD vs. HC *p* level
		Mean ± SD	Me (IQR 25%–75%)	Mean ± SD	Me (IQR 25%–75%)	
TG (mg/dL)	<100	117.3 ± 47.1	116.0 (83.8–144.0)	141.6 ± 25.7	137.7 (125.6–152.3)	<0.001
TC (mg/dL)	<190	141.7 ± 49.1	128.5 (112.0–168.3)	231 ± 38.6	234.7 (203.9–259.7)	<0.001
LDL (mg/dL)	<100	69.4 ± 41.0	61.0 (41.8–81.8)	85.4 ± 29.7	89.3 (63.4–103.0)	<0.001
HDL (mg/dL)	>45	53.8 ± 16.6	50.5 (41.0–64.3)	72.1 ± 17.6	70.8 (61.4–82.1)	<0.001
Non-HDL (mg/dL)	<100	88.8 ± 43.5	78.0 (62.0–104.5)	158.9 ± 43.5	159.3 (131.9–188.9)	<0.001
oxLDL (ng/dL)	-	361.9 ± 246.0	266.6 (228.6–415.8)	430.0 ± 416.4	290.0 (73.7–749.3)	0.879

Abbreviations: CAD, coronary artery disease; HC, healthy controls; TG, triglycerides; TC, total cholesterol; LDL, low-density lipoproteins; HDL, high-density lipoproteins; non-HDL cholesterol calculated by subtracting the HDL value from a TC; oxLDL, oxidized low-density lipoprotein; SD, standard deviation; Me, median; IQR, interquartile. *According to the American College of Cardiology 2023 [https://www.acc.org/Latest-in-Cardiology/Articles/2023/09/19/10/54/An-Update-on-Lipoprotein-a].

### Endothelium-specific variables

**FIGURE 1:**
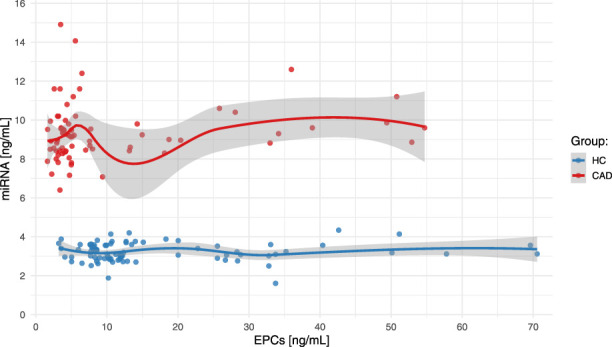
The distribution of endothelium-specific variables for miRNA and EPCs (red) coronary artery disease, micro-ribonucleic acids (blue) healthy control.

Significant differences in endothelium-specific variables, i.e., NO and 3NT revealed impaired endothelial secretory function in patients with coronary artery disease. The levels of NO, 3NT, eEPCs and EPCs were reduced in CAD group compared to HC (Table 5). Among CAD patients, no gender differences in NO levels were found, whereas healthy women displayed higher level of NO (330 ± 149 μmol/L) than healthy men (155 ± 26 μmol/L). 3NT levels were also significantly elevated in healthy women (0.950 ± 0.311 nmol/mL) than healthy men (0.708 ± 0.080 nmol/mL). The concomitant elevation of NO and 3NT suggest that NO is released from activated neutrophils and macrophages rather than from the endothelium. Furthermore, all women were at postmenopausal age, which excludes the involvement of the endothelium in NO production (Pruter et al., 2023). The relation of NO/3NT was recorded at r_s_ = 0.799 (*p* < 0.001) in CAD patients, and at r_s_ = 0.740 (*p* < 0.001) in HC. By contrast, miRNA levels were tree-fold higher in CAD patients. Circulating miRNAs in cardiovascular diseases could exhibit a prominent inflammatory character, which was also confirmed by high PIV values in CAD patients. The highest changes and values of AUC = 1.000 were observed for miRNA and 3NT (sensitivity 100%, specificity 100%), which is indicative of a very high probability of endothelial dysfunction once the cut-off value has been exceeded (Table 6). For all individuals, AUC = 1 with a cut-off at 5.37 ng/mL indicates a very high diagnostic utility of miRNA for clinical differentiation regardless of changes in EPCs (Figure 1). EPCs release a variety of factors, including microRNAs, which can promote vascular repair by stimulating endothelial cells and supporting regenerative processes (Zhang et al., 2014).

**TABLE 5:** Endothelium specific variables.

Variables	CAD *n* = 72		HC *n* = 80		CAD vs. HC *p* level
	Mean ± SD	Me (IQR 25%–75%)	Mean ± SD	Me (IQR 25%–75%)	
NO (µmol/L)	77.60 ± 50.15	53.58 (47.51–87.90)	290.64 ± 151.92	233.82 (163.59–386.77)	<0.001
3NT (nmol/mL)	0.576 ± 0.311	0.475 (0.397–0.601)	0.896 ± 0.301	0.816 (0.703–0.945)	<0.001
eEPCs (ng/mL)	10.42 ± 5.43	9.05 (7.59–10.40)	14.37 ± 9.42	11.00 (9.02–14.92)	<0.001
EPCs (ng/mL)	10.47 ± 13.26	4.72 (3.40–9.04)	17.32 ± 14.58	11.41 (8.38–24.86)	<0.001
miRNA (ng/mL)	9.36 ± 1.48	9.18 (8.46–9.84)	3.22 ± 0.48	3.19 (2.90–3.59)	<0.001

Abbreviations: CAD, coronary artery disease; HC, healthy comparison individual; NO, nitric oxide; 3NT, 3-nitrotyrosine; eEPCs, early endothelial progenitor cells; EPCs, endothelial progenitor cells; miRNA, micro ribonucleic acid; SD, standard deviation; Me, median; IQR, interquartile.

**TABLE 6:** The statistical characteristics of the ROC curve for the univariate logistic model for endothelium-specific variables in patients with coronary artery disease.

Variables	AUC	Cut-off value	Sensitivity (%)	Specificity (%)
NO (μmol/L)	0.954	119.66	98.8	81.3
3NT (nmol/mL)	1.000	68.96	100	100
EPCs (ng/mL)	0.772	7.76	86.2	73.6
miRNA (ng/mL)	1.000	5.37	100	100

Abbreviations: AUC, the area under the curve; cut-off value the optimal threshold value for clinical stratification.

## Discussion

Among the risk factors of cardiovascular diseases, increased visceral fat content can induce alterations in endothelial morphology and metabolism, which contributes to the development of coronary artery disease. Excess body weight through multiple pathways, including increased angiotensin, circulating blood volume and total peripheral resistance, leads to changes in endothelial function, increased dyslipidemia, and other inflammatory responses (Jahangir et al., 2014; Alkhawam et al., 2016). In our study sample, 40% of patients with CAD were obese (34.9 ± 4.1 kg/m^2^) and simultaneously demonstrated high levels of inflammation-exposed PIV (558 ± 563), whereas patients with normal BMI (23.3 ± 2.2 kg/m^2^) had low PIV (338 ± 142). CAD was associated with a markedly amplified innate immune signal. Total leukocyte counts were higher in CAD than in healthy individuals (7.63 ± 2.14 vs. 5.97 ± 1.63 ×10^3^/µL, *p* < 0.001), driven predominantly by neutrophilia (4.97 ± 1.90 vs. 3.27 ± 1.19 ×10^3^/µL, *p* < 0.001) and monocytosis (0.66 ± 0.25 vs. 0.52 ± 0.16 ×10^3^/µL, *p* < 0.001), with no between-group difference in platelet counts. This cellular pattern provides a mechanistic substrate for the substantially higher composite inflammatory indices in CAD, including a two-fold increase in PIV compared with HC. Obesity patients demonstrated impaired endothelial function manifested by reduced levels of NO and EPCs compared with normal-weight patients. Therefore, excess weight represents a main health challenge in the pathogenesis of CAD (Alkhawam et al., 2016).

Multiple studies have demonstrated that PIV is a strong inflammatory marker with prognostic value comparable to, or exceeding, that of previously established indices, and that it independently predicts CAD severity and adverse outcomes in both cardiovascular cohorts and the general population (Zhang et al., 2025; Kelesoglu et al., 2022; Liu et al., 2021). Higher PIV levels are linked to an increased risk of major adverse cardiovascular events, more severe coronary lesions, and poorer prognosis in patients with various forms of CAD, such as ST-segment elevation myocardial infarction and non-ST-segment elevation myocardial infarction (Cetinkaya et al., 2024). PIV was two-fold higher in coronary syndrome (477 ± 447) compared to healthy individuals (222 ± 166), which confirms it is a utility as a predictor of disease severity and adverse cardiovascular events. Another integrated marker, the neutrophil to high-density lipoprotein ratio, combines these two crucial factors into a single parameter, providing a more comprehensive assessment of cardiovascular risk than either neutrophil count or HDL alone. High Elevated NHR is independently associated with coronary artery disease and the severity of coronary lesions (Soehnlein and Döring, 2023). Although the CAD patients did not display dyslipidemia, NHR was twice as high compared to healthy controls, primarily due to increased neutrophil counts. Neutrophils, the most abundant circulating white blood cells, have not been underestimated in the pathology of cardiovascular disease but it is the evidence accumulated during the last decade that has firmly indicated their importance in cardiovascular diseases (Zhang et al., 2023). Activated neutrophils oxidize LDL to form oxLDL, releasing reactive oxygen species, thereby inducing endothelial damage (Smith et al., 2014). They may also mediate HDL oxidation and impair cholesterol efflux (Murphy et al., 2011). In contrast, HDL can inhibit neutrophil activation, adhesion, proliferation and migration (Murphy et al., 2011). Neutrophils are the primary cells in the acute inflammatory response and play an important role in the subsequent activation of monocytes and lymphocytes (Rosales, 2020). These mechanisms underpin the enhanced diagnostic and predictive value of NHR in cardiovascular disease (Soehnlein and Döring, 2023). Despite elevated neutrophil counts and NHR and lipoprotein-lipid profile elements were significantly lower in the patient group than in healthy individuals. The observed elevation of PIV is biologically consistent with a CAD phenotype characterized by coordinated activation of neutrophil- and monocyte-mediated inflammation. Importantly, the NHR signal remained higher in CAD despite lower circulating lipid and oxLDL measures in the patient group, which may partly reflect the effects of lipid-lowering and concomitant cardiovascular therapies. Therefore, the observed associations should be interpreted cautiously, as treatment-related effects may have influenced lipid parameters, inflammatory indices, EPCs and circulating miRNA levels.

In the presence of cardiovascular risk factors, both the number and functional capacity of endothelial progenitor cells are adversely affected, leading to impaired maintenance of endothelial integrity and homeostasis (Wojakowski et al., 2008; Yue et al., 2011). A fundamental feature of a healthy endothelium is its ability to generate adequate levels of nitric oxide, a key mediator of vascular tone, platelet inhibition, and anti-inflammatory signaling. ROS reduce NO bioavailability, thereby contributing to vascular dysfunction and the development of atherosclerotic disease. In particular, excess superoxide anion reacts with NO to form peroxynitrite (ONOO^−^), a highly reactive oxygen and nitrogen species that promotes oxidative stress and protein nitration (Förstermann et al., 2017). Although oxLDL tended to have lower values in coronary artery disease, it correlated with NO and 3NT, which confirms that oxLDL may influence endothelial functions, including processes such as proliferation, differentiation, apoptosis, mobilization, migration and senescence (Poznyak et al., 2021).

In the present study, both NO levels and its functional bioavailability, as reflected by 3-nitrotyrosine, were significantly reduced in patients with coronary artery disease compared with healthy controls. Despite these absolute differences, a strong association between NO and 3NT was observed in both groups, suggesting that nitrosative stress remains tightly linked to NO metabolism irrespective of disease status. Protein nitration has profound effects on protein structure and biological function and has been documented in numerous pathological conditions characterized by enhanced oxidative stress, serving as a recognized clinical marker of cardiovascular diseases (Daiber et al., 2021). Indeed, tyrosine nitration of key plasma proteins, including fibrinogen, apolipoprotein A-1, and apolipoprotein B-100, has been demonstrated in patients with CAD, indicating that functional alterations of nitrotyrosine-modified proteins may directly contribute to endothelial dysfunction and atherogenesis (Jiang et al., 2022). As NO is the principal endogenous nitrosative mediator, its reduced bioavailability plays a central role in endothelial dysfunction and promotes adverse arterial remodeling through multiple interrelated mechanisms (Daiber and Münzel, 2012). Consequently, the alterations in NO and 3NT observed in our cohort may reflect a sustained nitrosative imbalance that predisposes CAD patients to adverse cardiovascular events.

Restoration of endothelial integrity following injury requires timely suppression and resolution of inflammation; processes closely associated with the mobilization and activation of endothelial progenitor cells (Zhang et al., 2014). EPC mobilization from the bone marrow is largely driven by inflammatory signaling pathways (Ti et al., 2009). This relationship has been previously examined by Morishita et al. (2012), who demonstrated that EPC dynamics are closely linked to markers of inflammation and oxidative stress in patients with cardiovascular diseases. Consistent with these findings, our study revealed a marked reduction in circulating EPCs, with approximately two-fold lower levels in CAD patients compared with healthy individuals. This decrease reflects impaired endothelial repair capacity and a diminished ability of EPCs to proliferate, differentiate, and mature into functional endothelial cells in the context of coronary artery disease (Yoder, 2012).

An additional factor exacerbating endothelial dysfunction in our cohort was excess body weight, observed in 40% of CAD patients. Obesity-related alterations of the endothelial glycocalyx, combined with chronic low-grade inflammation originating from adipose tissue, contribute to a pro-inflammatory and pro-thrombotic vascular milieu (Li et al., 2021). These mechanisms further compromise endothelial function and may accelerate atherosclerotic progression. Notably, accumulating evidence indicates that cardiovascular risk associated with endothelial dysfunction can be attenuated through effective modulation of inflammatory pathways and targeted management of obesity (Kwaifa et al., 2020).

Endothelial dysfunction, a fundamental step in the development and progression of atherosclerosis and CAD, is increasingly recognized as being directly or indirectly regulated by microRNA-mediated mechanisms (Mansour et al., 2025; Ait-Aissa et al., 2020). Circulating microRNAs reflect the activation status of immune and endothelial cells, and have therefore emerged as potential biomarkers of both cellular activation and tissue injury in response to cardiovascular risk factors (Zhang et al., 2014; Zampetaki et al., 2012; Coco et al., 2019). Importantly, circulating miRNA profiles may also mirror changes occurring at the myocardial and vascular wall level, including processes related to myocardial remodeling and endothelial injury, providing additional insight into the molecular mechanisms underlying cardiovascular disease (Menezes Junior et al., 2023; Moreau et al., 2021). A growing body of experimental and clinical evidence indicates that specific miRNAs are directly involved in the post-transcriptional regulation of immunity-related genes. Pro-inflammatory miRNAs such as miR-155, miR-21, miR-34a, miR-33a/b, and miR-92a have been consistently implicated in endothelial injury, macrophage activation, neutrophil recruitment, and atherosclerotic plaque destabilization. In contrast, miR-146a, miR-223, miR-181b, and miR-126 exert vasculoprotective effects by promoting endothelial repair and angiogenesis (Leonard et al., 2024; Olivieri et al., 2021). Notably, several miRNAs have been shown to correlate with hematologic inflammatory indices such as the neutrophil-to-lymphocyte ratio (NLR) and platelet-to-lymphocyte ratio (PLR), suggesting that alterations in circulating miRNA levels may reflect systemic inflammatory status through modulation of innate immune cell activity (Zhu et al., 2013).

In our study, total circulating miRNA concentrations were markedly elevated in patients with CAD compared to healthy controls, with median values of 9.18 ng/mL vs. 3.19 ng/mL (*p* < 0.001), representing a three-fold increase. This pronounced separation between groups suggests that elevated miRNA levels are not merely an epiphenomenon, but they reflect a biologically meaningful state of heightened immune activation and endothelial injury characteristic of atherosclerotic disease. It is noteworthy that CAD patients in our study also exhibited significantly higher PIV and NHR values, the indices driven primarily by increased neutrophil and monocyte counts. Given the established role of neutrophils and monocytes as major cellular sources and targets of inflammation-associated miRNA signaling, the parallel elevation of circulating miRNAs and composite inflammatory indices may indicate a relationship between miRNA dysregulation and innate immune activation in CAD.

From a pathophysiological perspective, increased circulating miRNA levels in CAD may reflect processes associated with vascular inflammation and endothelial dysfunction. Previous experimental and clinical studies have demonstrated that activated neutrophils and monocytes can release specific miRNAs either freely or within extracellular vesicles, which may contribute to inflammatory signaling, impair endothelial nitric oxide bioavailability, and promote vascular remodeling (Li et al., 2025). Conversely, endothelial injury itself can stimulate the release of miRNAs into the circulation (Silva et al., 2025). Collectively, these observations provide a plausible explanation for the observed associations between miRNA levels, PIV, NHR, and established markers of endothelial dysfunction in our cohort.

Our findings provide further evidence of complex interactions between circulating miRNAs, inflammation-related indices, and endothelial dysfunction in coronary artery disease. The results suggest that total circulating miRNAs, particularly when analyzed together with endothelial-related parameters, may represent promising biomarkers for clinical differentiation and characterization of endothelial dysfunction in CAD patients, although the potential influence of ongoing cardiovascular therapies should be taken into consideration. These findings support the potential use of miRNAs as biomarkers; however, they should not yet be interpreted as evidence of their established role as clinical diagnostic tools.

## Limitations

Several limitations of the present study should be acknowledged. First, the relatively limited sample size and the pronounced biological separation observed between CAD patients and healthy controls may have contributed to the exceptionally high diagnostic performance of circulating total miRNAs, including the observed AUC values of 1.0. Therefore, these findings should be interpreted with caution and considered exploratory until confirmed in larger, independent cohorts and external validation studies. Second, the study was conducted in a single-center cohort, which may limit the generalizability of the findings to broader patient populations. Third, due to the cross-sectional design of the study, no causal relationships can be established between circulating miRNA levels, inflammatory activation, endothelial dysfunction, and CAD progression. Consequently, although circulating miRNAs demonstrated strong discriminatory potential, the present study does not allow definitive conclusions regarding their long-term prognostic utility or predictive value for future cardiovascular outcomes.

## Data Availability

The raw data supporting the conclusions of this article will be made available by the authors, without undue reservation.
